# Childhood internalising symptoms at ages 3 and 6: a meta‐analysis of epigenome‐wide associations from cord and peripheral blood

**DOI:** 10.1111/jcpp.70186

**Published:** 2026-06-16

**Authors:** Laura Schellhas, Mannan Luo, Janine F. Felix, Christian M. Page, Mona Bekkhus, Marcus R. Munafò, Luisa Zuccolo, Alexandra Havdahl, Charlotte A.M. Cecil, Gemma C. Sharp

**Affiliations:** ^1^ School of Psychological Science University of Bristol Bristol UK; ^2^ MRC Integrative Epidemiology Unit University of Bristol Bristol UK; ^3^ Department of Child & Adolescent Psychiatry/Psychology, Erasmus MC University Medical Center Rotterdam Rotterdam The Netherlands; ^4^ The Generation R Study Group, Erasmus MC University Medical Center Rotterdam Rotterdam The Netherlands; ^5^ Department of Pediatrics, Erasmus MC University Medical Center Rotterdam Rotterdam The Netherlands; ^6^ Division for Mental and Physical Health, Department of Physical Health and Aging Norwegian Institute of Public Health Oslo Norway; ^7^ Department of Psychology, PROMENTA Research Centre University of Oslo Oslo Norway; ^8^ Research Department Lovisenberg Diaconal Hospital Oslo Norway; ^9^ Health Data Science Centre Fondazione Human Technopole Milan Italy; ^10^ Division of Public Health and Prevention, PsychGen Centre for Genetic Epidemiology and Mental Health Norwegian Institute of Public Health Oslo Norway; ^11^ School of Psychology University of Exeter Exeter UK

**Keywords:** PACE consortium, internalising problems, DNA methylation

## Abstract

**Background:**

Pre‐ and postpartum environments and genetic effects influence childhood internalising problems, which increase depression risk. DNA methylation (DNAm) may capture some of these effects. We therefore investigated associations between child blood DNAm and internalising problems.

**Methods:**

We meta‐analysed probe and region‐level epigenome‐wide association studies using data from 3 European birth cohorts (ALSPAC, MoBa, Generation R; analytic *N* = 1,121–3,011) from the Pregnancy And Childhood Epigenetics (PACE) Consortium. DNAm was assessed at birth (cord blood) and age 6 years (peripheral blood). Internalising problems (ages 3 and 6) were reported by mothers using the Child Behaviour Checklist or Strengths and Difficulties Questionnaire. Models were adjusted for age at DNAm assessment, 20 surrogate variables, estimated cell proportions, maternal education, age, smoking and, in secondary analysis, maternal anxiety and depression. Public databases were searched for mental health associations with top CpG sites and regions.

**Results:**

No significant probe‐level associations were found between cord‐ or peripheral‐blood DNAm and internalising problems. In region‐level analyses, two differentially methylated regions (DMRs) in cord blood were associated with internalising problems at age 3 (annotated to *STK32C*, *MIR886*) and one at age 6 (*PFKFB2*). At age 6, peripheral‐blood analyses identified two DMRs (*C10orf26* (*WBP1L*), *FAM125A*). Several genes showed prior associations with psychiatric phenotypes, including depression.

**Conclusions:**

The higher‐powered regional‐level analyses revealed more associations than probe‐level. Among others, we identified a region annotated to *STK32C* that has previously been linked to adolescent depression. Larger samples and refined phenotyping are needed to clarify the role of DNAm in internalising problems.

## Introduction

Childhood internalising problems reflect a broad, dimensional liability to inwardly directed distress (e.g. anxiety, low mood, somatic complaints). In early childhood, symptom profiles are often diffuse and co‐occur, and dimensional/hierarchical models emphasise that higher‐order internalising liability accounts for substantial covariance among early emotional symptoms (Kotov et al., 2017; Stevenson, 2023). Internalising problems are clinically important, as they increase the risk for later anxiety and depressive disorders and are associated with poorer academic performance, peer relationships and life satisfaction (Ingeborgrud et al., 2023; Sallis et al., 2019; Whalen, Sylvester, & Luby, 2017). For example, parent‐reported anxiety and depressive symptoms at age 3 predicted anxiety disorder and/or depression at age 8 (OR = 1.30 and 1.80, respectively) (Ingeborgrud et al., 2023). Clarifying how internalising difficulties emerge early in emotion‐regulation development, and which factors shape this trajectory, is therefore crucial for informing prevention before more severe and differentiated psychopathology consolidates (Stevenson, 2023). Despite the clinical need to detect internalising problems early, symptoms are often missed in routine care, in part because they present as quiet, ‘intropunitive’ distress rather than disruptive behaviour and therefore tend to be under‐recognised by parents, teachers and clinicians (Koning et al., 2019; Splett et al., 2019; Tandon, Cardeli, & Luby, 2009). This under‐recognition motivates interest in complementary indicators of risk that could help clarify how vulnerability develops and, in principle, augment symptom‐based identification‐particularly for children whose distress is less visible.

Both genetic and environmental factors contribute to risk for internalising psychopathology (Kwong et al., 2019). Polygenic liability for neuroticism—a transdiagnostic risk factor for later anxiety and depression—has been linked to higher levels and altered developmental trajectories of internalising problems from infancy through late childhood (Costantini et al., 2023), indicating that common genetic risk is already expressed in early emotional–behavioural profiles. Twin studies indicate moderate heritability (~40%) and substantial shared genetic liability for childhood anxiety and depression (Eley, 1999; Gottschalk & Domschke, 2017; Polderman et al., 2015), whereas SNP‐based heritability estimates from GWAS are lower (≈26%–31% for anxiety; ≈8%–9% for depression) (Howard et al., 2019; Kwong et al., 2019; Major Depressive Disorder Working Group of the Psychiatric Genomics Consortium, 2025). This pattern highlights that common variants capture only part of genetic liability; motivating investigation of how environmental exposures may become biologically embedded and contribute to risk. Observational evidence further implicates prenatal and early postnatal exposures, such as maternal tobacco use during pregnancy, early trauma or abuse, and parental psychopathology to be associated with internalising problems (Ahmadzadeh et al., 2021; Duko, Ayano, Pereira, Betts, & Alati, 2020; LeMoult et al., 2020). Together, these findings motivate investigation of molecular mechanisms that integrate genetic liability with early environmental exposures.

Epigenetic processes, particularly DNA methylation (DNAm), provide a plausible molecular correlate of such biological embedding. DNAm, the most widely studied epigenetic modification, regulates gene activity without altering DNA sequence via the addition of methyl groups to cytosine–phosphate–guanine (CpG) sites (Bird, 2007). DNAm is sensitive to prenatal and early‐life environments and is especially dynamic during early childhood, a period of marked neurobiological and epigenomic plasticity (Cortessis et al., 2012; Perera & Herbstman, 2011). Studying internalising symptoms during this developmental window is therefore well suited to epigenetic investigation. Based on this, we hypothesised that early‐life DNAm—measured at birth and in childhood—would show reproducible associations with early internalising problems. Importantly, however, DNAm‐phenotype associations do not by themselves establish mediation or causality, and large‐scale genetic studies of blood DNAm highlight that causal interpretations can be difficult to support for many DNAm‐trait links (Min et al., 2021). We therefore treat DNAm here as a potential source of (i) candidate biological correlates that could motivate mechanistic follow‐up where signals and designs permit and/or (ii) candidate biomarkers of risk to be evaluated in future predictive work.

To date, EWAS relevant to child internalising problems have more often examined DNAm correlates of risk exposures (e.g. maltreatment) than DNAm correlates of internalising symptoms themselves (Dunn et al., 2019; Juruena, Gadelrab, Cleare, & Young, 2021; Parade et al., 2021). The relatively small number of symptom‐focused EWAS in youth have reported inconsistent findings across ages and tissues and have generally been underpowered (typically *N* < 300 across blood/buccal/saliva samples; Dempster et al., 2014; Kallak et al., 2022; Weder et al., 2014). A larger study examined placental DNAm in relation to child internalising problems (*N* ≈ 441; Nakamura et al., 2023), but placental DNAm primarily reflects intrauterine influences rather than the child's systemic methylome. This literature contrasts with stronger aggregation efforts in adulthood, where larger EWAS meta‐analyses of depressive symptoms have achieved substantially bigger sample sizes (*N* = 7,948) and more stable signal detection, illustrating the scale likely needed for robust discovery (Story Jovanova et al., 2018). Together, these patterns highlight the need for well‐powered, harmonised analyses of child internalising outcomes timed to development (Cecil, Neumann, & Walton, 2023; Mansell et al., 2019; Non, 2021).

The present study addresses these gaps by meta‐analysing EWAS from three European pregnancy and birth cohorts in the Pregnancy And Childhood Epigenetics (PACE) Consortium (maximum analytic *N* = 3,011). We examine internalising problems at ages 3 and 6 years and DNAm at birth (cord blood; prospective) and at age 6 (peripheral blood; cross‐sectional). The prospective birth‐DNAm analyses support temporal ordering; the age‐6 DNAm analyses are cross‐sectional and are interpreted as same‐age associations, which cannot establish a temporal sequence between DNAm and symptoms. Across analyses, we meta‐analysed DNAm—internalising problems associations across cohorts and time points—providing an essential first step for subsequent causal follow‐up or utility as risk biomarkers.

## Methods

### Participating cohorts

Three independent birth cohorts contributed data on offspring cord‐blood DNAm and internalising problems for this study: One UK‐based cohort (the Avon Longitudinal Study of Parents and Children, ALSPAC; *N* = 739) (Boyd et al., 2013; Fraser et al., 2013), one Dutch cohort (Generation R; *N* = 793) (Kooijman et al., 2016; Kruithof et al., 2014) and two datasets from a Norwegian cohort (the Norwegian Mother, Father and Child Cohort, MoBa1, *N* = 998 and Moba2, *N* = 481) (Brandlistuen et al., 2025; Magnus et al., 2016). Recruitment periods differed among cohorts, from the early 1990s (ALSPAC), 2002–2006 (Generation R) and 1999–2008 (MoBa). At the time of analysis, internalising problems at age 6 were only accessible in ALSPAC and Generation R; thus, MoBa data contributed exclusively to the prospective analysis of internalising problems at age 3. Ethical approval was obtained by local ethics committees, and informed consent for the use of data was obtained from all participants. We excluded multiple pregnancies (e.g. twins) and siblings so that each mother was only represented once in the datasets. For more details about the individual cohorts, please refer to Appendices S1–S3.

### Measures

#### DNA methylation

DNAm data were sampled using the lllumina Infinium® HumanMethylation450 (486,425 probes). Cohorts assessed methylation data individually, using their own laboratory methods, quality control and normalisation. Cord‐blood and peripheral‐blood DNAm were assessed through normalised beta values. Probes on SNPs, probes that cross‐hybridised according to Chen and colleagues (Chen et al., 2013), and probes on sex chromosomes were excluded (Appendices S1–S3).

#### Childhood internalising problems

Generation R and MoBa assessed child internalising problems by maternal report on the internalising subscale of the Child Behaviour checklist (CBCL‐1½‐5) (Achenbach & Rescorla, 2000). MoBa1 and MoBa2 used a shortened scale (subset of 9 items) of the full internalising scale. In ALSPAC, internalising problems were assessed using maternal report on the emotional symptoms subscale of the Strengths and Difficulties Questionnaire (SDQ) (Goodman, 1997). Internalising problems scores were *z*‐standardised to ensure compatibility of the scales used in the different cohorts. More information on the measures and their comparability can be found in Appendices S1–S3.

#### Covariates

Because DNAm is sensitive to technical, biological and environmental variation, we prioritised covariate control to reduce spurious CpG associations (Jones, Moore, & Kobor, 2018). At the same time, we sought to avoid the risk of over‐adjustment when variables that may lie on the etiologic pathway are included. We therefore used a hierarchical covariate strategy that first accounts for technical and biological factors (cell composition) and then evaluates how estimates change when adding correlated prenatal/familial covariates, providing transparent context for interpreting any detected signals. All EWAS were run in the following hierarchical covariate sequence, identically for the prospective (cord blood) and cross‐sectional (peripheral blood) analyses:

*Minimally adjusted (technical + cell composition)*: included 20 surrogate variables derived via surrogate variable analysis (SVA) to account for residual, unmeasured non‐biological variation and latent technical/batch structure in microarray DNAm (e.g. plate/array, position, DNA input and processing date) (Leek, Johnson, Parker, Jaffe, & Storey, 2012), plus estimated blood cell‐type proportions (Houseman method; cord‐blood reference for birth DNAm (Gervin et al., 2016); peripheral‐blood reference for childhood DNAm (Reinius et al., 2012)).

*Main model (covariate adjusted)*: Minimally adjusted model + child age at DNAm assessment + maternal age, maternal education and maternal smoking during pregnancy. Maternal smoking was assessed via self‐report across cohorts rather than methylation‐derived scores, to avoid adjusting for variables derived from the same DNAm matrix and thus reduce the risk of over‐adjustment or attenuation of biologically meaningful associations (Appendix S4).

*Sensitivity model (covariate plus maternal anxiety/depression adjusted)*: Main model + maternal prenatal anxiety and depression (*z*‐standardised within cohort). This model was used to assess robustness to correlated prenatal/familial factors.

*Secondary analyses (sex stratified)*: Main model estimated separately in females and males.

We decided to adjust for maternal anxiety/depression in a separate sensitivity model (z‐standardised within cohort) because maternal anxiety/depression may lie on upstream exposure → DNAm → outcome pathways and thus was not included in the main models to avoid over‐adjustment. The sensitivity analysis targets the DNAm–internalising association conditional on maternal anxiety/depression, probing robustness to familial liability, correlated exposures and shared‐informant variance. We also conducted sex‐stratified secondary analyses, given known sex differences in DNAm that can persist even after restricting to autosomes and removing cross‐reactive probes (Yousefi et al., 2015).

### Statistical analyses

#### Cohort‐specific statistical analyses

Outliers were removed from the methylation data using the Tukey method (values < (25th percentile − 3*interquartile range) and values > (75th percentile + 3*interquartile range)) (Tukey, 1977). For the probe‐level analysis, we ran a linear regression at each CpG with offspring DNAm as the exposure and internalising problems in childhood as the outcome using the R‐package Limma (Ritchie et al., 2015). Probes were annotated to the human reference genome version 19, build 37 using the annotation data available from the R‐package meffil (Suderman, Hemani, & Min, 2019). We also conducted a regional analysis, which has higher statistical power than probe‐level analyses, in each cohort using the dmrff R‐package (Suderman et al., 2018).

#### Meta‐analyses

Overall, three different EWAS meta‐analyses were run using a probe‐level and a regional analysis approach:

1. Prospective analysis age 3: Internalising problems age 3 ~ Cord‐blood DNAm (cohorts: ALSPAC, MoBa, Generation R; *N* = 3,011)
2. Prospective analysis age 6: Internalising problems age 6 ~ Cord‐blood DNAm (cohorts: ALSPAC, Generation R; *N* = 1,601)
3. Cross‐sectional analysis age 6: Internalising problems age 6 ~ Peripheral DNAm age 6 (cohorts: ALSPAC, Generation R; *N* = 1,121)

### Probe‐level analysis

Quality checks on the meta‐analysis results were conducted (QQ‐ and correlation plots) (Sharp et al., 2021; Van der Most, Küpers, Snieder, & Nolte, 2017). Results of the probe‐level analysis were meta‐analysed with fixed effect estimates, weighted by the inverse of the variance using METAL (Willer, Li, & Abecasis, 2010). To account for multiple testing, *p*‐values were adjusted using a 5% false discovery rate (FDR) (Benjamini & Hochberg, 1995) and values <.05 were considered as evidence for statistical significance. FDR correction is recommended for large‐scale, correlated EWAS data and offers appropriate error control with greater power than Bonferroni correction (Rakyan, Down, Balding, & Beck, 2011; Shaffer, 1995). A leave‐one‐cohort‐out analysis was performed on the CpGs with the strongest evidence for a statistical association (as indicated by the smallest *p*‐value) using the R‐package *metafor* (Viechtbauer, 2010). A change in direction of the estimate, moved toward the null by more than 20%, or a confidence interval that included 0 after removal of a single cohort were considered as an indication for results being driven by a single cohort. A shadow meta‐analysis was conducted independently by researchers at Generation R.

### Differentially methylated regions (DMR) analysis

The meta‐analysis of the DMR cohort results was conducted using the *dmrff.meta* function within the dmrff R‐package, which uses an inverse‐variance weighted fixed‐effects approach (Suderman et al., 2018). A region was defined as having at least two CpGs that are less than 500 bp apart show the same direction of effect and a Bonferroni‐adjusted *p*‐value (*p*_Bonferroni_) < .05 (Shaffer, 1995).

### Database look‐up

To contextualise findings from the probe and DMR analyses, we consulted publicly available databases: GeneCards (https://www.genecards.org/), NHGRI‐EBI GWAS catalogue (GWAS catalogue; https://www.ebi.ac.uk/gwas/) and EWAS catalogue (http://www.ewascatalog.org/) and manually screened for mental health phenotypes. We defined mental health phenotypes as psychiatric diseases or symptoms of psychiatric diseases (excluding substance abuse). Childhood abuse was also included, given its frequent investigation in relation to DNAm (Dunn et al., 2019; Juruena et al., 2021; Mehta et al., 2023; Parade et al., 2021) and its established role as a risk factor for later depression and anxiety (Gardner, Thomas, & Erskine, 2019; LeMoult et al., 2020; McKay et al., 2022).

## Results

### Sample characteristics

Overall, offspring and maternal characteristics were similar across the cohorts (Table 1). The sample of the prospective meta‐analysis at age 3 contained more mothers with at least a high school diploma (69%) than without, and this pattern was consistent across cohorts, except for ALSPAC, where having a high school diploma or not was approximately equally distributed (Table 1). On average, 11% of mothers in the samples smoked in the third trimester or throughout pregnancy (Table 1). Across samples, mothers were on average 31 years of age at the birth of the study child (range: 29.8–32.3 years; Table 1). Female‐ and male‐sex children showed similarly low levels of internalising problems across cohorts (Table 1). Across cohorts and time points, there was evidence for a statistically significant positive correlation between maternal anxiety and depressive symptoms during pregnancy and offspring internalising problems, yet the magnitude of associations was rather small (correlation range: .13–.23; Appendix S5, Table S1). As expected, there was evidence for internalising problems at age 3 being positively associated with internalising problems at the age of 6, but only of moderate size, indicating that internalising problems change from age 3 to 6 (correlation range: .39–.56; Appendix S5, Table S1).

**Table 1:** Sample characteristics

Cohort	*n*	*M* age in months (*SD*)^a^	*M* gestational age in weeks (*SD*)	*N* (~%) Female	Item mean (*SD*) of internalising subscale^b^	Item mean (*SD*) of internalising subscale^b^ – Female sex	Item mean (*SD*) of internalising subscale^b^ – Male sex	Maternal education ≥ *high school diploma* (*n* (%))	Maternal age (Mean, *SD*)	Maternal continued smoking (*N* (%))	Maternal anxiety/depression during pregnancy (Mean (*SD*))^b^
Internalising problems age 3 ~ Cord‐blood DNAm											
ALSPAC	739	47.59 (0.85)	39.55 (1.49)	377 (51%)	0.26 (0.27)	0.28 (0.04)	0.25 (0.28)	372 (50%)	29.75 (4.32)	79 (11%)	0.63 (0.46)
Generation R	793	36.36 (0.84)	40.26 (1.40)	400 (50%)	0.12 (0.10)	0.12 (0.11)	0.12 (0.10)	560 (71%)	32.06 (3.99)	83 (11%)	0.13 (0.22)
Moba1	998	47.59 (0.85)	39.97 (1.55)	499 (50%)	0.24 (0.42)	0.25 (0.22)	0.23 (0.21)	838 (84%)	29.97 (4.30)	146 (15%)	0.23 (0.35)
Moba 2	481	28.93 (16.38)	39.95 (1.45)	212 (44%)	0.24 (0.22)	0.25 (0.24)	0.23 (0.20)	391 (81%)	29.90 (4.29)	43 (9%)	0.23 (0.34)
Mean or total	3,011	41.67 (1.18)	39.92 (1.50)	1,445 (48%)	–	–	–	2,161 (72%)	30.52 (4.21)	351 (12%)	–
Internalising problems age 6 ~ Cord‐blood DNAm											
ALSPAC	709	81.20 (1.00)	39.54 (1.51)	356 (50%)	0.25 (0.28)	0.27 (0.27)	0.24 (0.29)	366 (52%)	29.92 (4.35)	74 (10%)	0.90 (0.47)
Generation R	892	70.92 (3.6)	40.25 (1.40)	448 (50%)	0.14 (0.14)	0.13 (0.13)	0.14 (0.14)	578 (65%)	31.78 (4.21)	103 (12%)	0.13 (0.26)
Mean or total	1,601	80.29 (1.43)	39.91 (1.49)	804 (50%)	–	–	–	944 (60%)	30.99 (4.27)	177 (11%)	–
Internalising problems age 6 ~ Peripheral‐blood DNAm at age 6											
ALSPAC	747	81.22 (1.06)	‐	372 (50%)	0.27 (0.29)	0.27 (0.27)	0.26 (0.30)	384 (51.4%)	29.89 (4.33)	80 (11%)	0.60 (0.49)
Generation R	374	71.28 (4.32)	–	195 (52%)	0.12 (0.13)	0.12 (0.12)	0.13 (0.14)	264 (70.6%)	32.31 (3.90)	40 (11%)	0.11 (0.22)
Mean or total	1,121	80.93 (1.28)	–	572 (51%)	–	–	–	648 (57.8%)	30.81 (4.17)	120 (11%)	–

In the Total row, means were weighted by the inverse variance for each cohort. *M*, mean.

^a^ Age at DNAm assessment.

^b^ As each cohort used different scales (Appendix S4), it was not possible to generate a total sample mean. Item means were calculated by dividing the total score by the number of items. DNAm, DNA methylation.

### Quality control checks

The meta‐analyses quality control checks did not indicate evidence for systematic confounding (Appendix S5; Figures S1–S11). As expected, correlations between the regression coefficients across models were higher when DNAm was assessed in the same tissue (cord blood *r* = .282) than across tissues (cord and peripheral blood *r* = .003; Appendix S5, Figure S3). Comparison across the hierarchical covariate structures showed similar patterns of findings (Appendix S5, Table S2), suggesting that results were not artefacts of technical, cellular or correlated prenatal/familial confounding.

### Probe‐level meta‐analyses

#### Prospective probe‐level analyses in cord blood

The main analyses revealed no statistically significant association between DNAm in cord‐blood and internalising problems at ages 3 or 6 after correcting for multiple testing (Table 2). These results remained consistent after adjusting the main model for maternal anxiety and depression. In the secondary sex‐stratified analyses, only the female‐specific analyses showed statistical evidence for associations between DNAm at one CpG site in cord blood and internalising problems at age 6 (cg26668632, nearest gene *IFNGR1*; estimate = −8.76, 95% CI: −11.78 to −5.74, *p*‐value = 1.42 × 10^−8^). The direction of effect for cg26668632 was consistent in the female‐ and male‐sex strata (Appendix S5, Table S3), with a larger magnitude in the female than male subgroup and did survive the leave‐one‐out analysis (Appendix S5, Figure S12). This pattern was consistent whether using FDR or Bonferroni thresholds (Appendix S5, Table S2).

**Table 2:** A summary of results of each EWAS model from the probe‐level analysis

Model	*N* CpGs with FDR‐corrected *p*‐value < 0.05	*N* CpGs (%) surviving leave‐one‐out analysis	Meta‐analysis sample size	Genomic inflation factor (λ)^a^
Prospective analysis: Internalising problems age 3 ~ Cord‐blood DNAm (*N* = 3,011)				
All offspring (minimally adjusted)^b^	0	n.a.	3,011	1.01
All offspring (adjusted for covariates)	0	n.a.	3,011	1.00
Female‐sex offspring (adjusted for covariates)	0	n.a.	1,452	1.02
Male‐sex offspring (adjusted for covariates)	0	n.a.	1,559	0.99
All offspring (adjusted for covariates and maternal anx./dep)	0	n.a.	2,987	1.00
Prospective analysis: Internalising problems age 6 ~ Cord‐blood DNAm (*N* = 1,601)				
All offspring (minimally adjusted)^b^	0	n.a.	1,601	1.02
All offspring (adjusted for covariates)	0	n.a.	1,601	1.03
Female‐sex offspring (adjusted for covariates)	1	1 (100%)	804	1.02
Male‐sex offspring (adjusted for covariates)	0	n.a.	797	1.06
All offspring (adjusted for covariates and maternal anx./dep)	0	n.a.	1,601	1.03
Cross‐sectional analysis: Internalising problems age 6 ~ Childhood DNAm age 6 (*N* = 1,121)				
All offspring (minimally adjusted)^b^	0	n.a.	1,601	0.99
All offspring (adjusted for covariates)	0	n.a.	1,136	0.98
Female‐sex offspring (adjusted for covariates)	2	1 (50%)	572	1.02
Male‐sex offspring (adjusted for covariates)	0	n.a.	564	0.94
All offspring (adjusted for covariates and maternal anx./dep)		n.a.	1,136	0.98

^a^ The genomic inflation factor (λ) estimates the extent of bulk inflation of EWAS *p*‐values and the excess false positive rate. 1 = no inflation; > 1 some evidence of inflation.

^b^ Only adjusted for estimated cell counts and 20 surrogate variables. Covariates: maternal age, maternal smoking, maternal education, offspring age, estimated cell counts and 20 surrogate variables.

#### Cross‐sectional probe‐level analysis in peripheral blood

No association between DNAm in peripheral blood age 6 and internalising problems at the age of 6 was found in the main model and the model additionally adjusted for maternal anxiety and depression (Table 2). Of the sex‐stratified analyses, only the female‐specific model found DNAm at two CpGs in peripheral blood in childhood (cg07283896; estimate = 6.10, 95% CI: 4.20 to 8.00; *p*‐value = 3.20 × 10^−10^; cg08884410; estimate = 8.59, 95% CI: 5.41 to 11.77, *p*‐value = 1.11 × 10^−7^; no annotated genes; Table S3) to be associated with internalising problems at age 6. This pattern was consistent whether using FDR or Bonferroni thresholds (Appendix S5, Table S2). Of those internalising problems‐associated CpGs, only cg08884410 survived the leave‐one‐out analysis (Appendix S5, Figures S13 and S14). The association with internalising problems at cg08884410 showed a consistent direction of effect across time points and cohorts in peripheral blood of female‐sex children (Appendix S5, Table S3). Only cg07283896 showed a different direction of effect across the sex strata and time points (Appendix S5, Table S3). The female subgroup had slightly more children (+8), while the male subgroup showed greater variability in effect sizes across studies (*I* ^2^ = 73.9% for males; *I* ^2^ = 0% for females).

### Differentially methylated regions meta‐analysis

An overview of the DMR meta‐analyses results can be found in Table 3.

**Table 3:** Result overview of the DMR meta‐analyses

	Regions	Estimate (*SE*)	*p*‐Value	Annotated genes
Prospective analysis: Internalising problems age 3 ~ Cord‐blood DNAm				
Main model (*n* = 3,011)	Chr10: 134045514–134045913	5.08 (0.88)	9.230 × 10^−09^	*STK32C*
	Chr5: 135415693–135416613	−4.80 (0.87)	3.948 × 10^−08^	*MIR886*
Main model + adjustment maternal anxiety/depression (*n* = 2,987)	Chr10: 134045514–134045913	5.37 (0.88)	1.165 × 10^−09^	*STK32C*
Main model stratified for male sex (*n* = 1,559)	–	–	–	*–*
Main model stratified for female sex (*n* = 1,452)	Chr5: 135415693–135416613	−8.54 (1.28)	2.870 × 10^−11^	*MIR886*
	Chr7: 95025611–95026095	39.50 (7.18)	3.819 × 10^−08^	*PON3*
	Chr6: 32120783–32121055	−17.66 (3.24)	4.868 × 10^−08^	*PPT2; PRRT1*
Prospective analysis: Internalising problems age 6 ~ Cord‐blood DNAm				
Main model (*n* = 1,601)	Chr1: 207226769–207226830	196.12 (34.04)	8.377 × 10^−9^	*PFKFB2*
Main model + adjustment maternal anxiety/depression (*n* = 1,601)	Chr1: 207226769–207226830	196.05 (33.67)	5.805 × 10^−9^	*PFKFB2*
Main model stratified for male sex (*n* = 797)	Chr14: 105190337–105190477	648.61 (108.59)	2.330 × 10^−9^	*ADSSL1*
Main model stratified for female sex (*n* = 804)	Chr13: 36050844–36050889	−79.94 (14.73)	5.775 × 10^−08^	*MIR548F5; NBEA;MAB21L1*
	Chr11: 35441311–35441777	−456.04 (84.32)	6.359 × 10^−08^	*SLC1A2*
Cross‐sectional analysis: Internalising problems age 6 ~ Childhood DNAm age				
Peripheral‐blood DNAm: Main model (*n* = 1,136)	Chr10: 104535961–104536121	−55.48 (8.83)	3.302 × 10^−10^	*C10orf26*
	Chr19:17531148–17531370	154.00, (28.79)	8.832 × 10^−8^	*FAM125A*
Peripheral‐blood DNAm: Main model + adjustment maternal anxiety/depression (*n* = 1,136)	Chr2:14772562–14772568	5.37 (14.21)	8.049 × 10^−8^	*FAM84A*
Peripheral‐blood DNAm: Main model stratified for male sex (*n* = 564)	Chr7:27170412–27170994	−18.33 (2.65)	4.814 × 10^−12^	*HOXA4*
Peripheral‐blood DNAm: Main model stratified for female sex (*n* = 572)	Chr10:115934483–115934488	535.78 (92.54)	7.046^−9^	*MIR2110;C10orf118*
	Chr13:31039916–31040215	219.62 (38.51)	1.178 × 10^−8^	*HMGB1*
	Chr12:117627133–117627450	85.11 (15.81)	7.299 × 10^−8^	*FBXO21*
	Chr1:160054219–160054321	52.88 (9.99)	1.188 × 10^−7^	*KCNJ9*

Main model is adjusted for offspring age at DNAm assessment, 20 surrogate variables, estimated cell proportions, maternal education, age and smoking during pregnancy. DMR, differentially methylated region.

#### Prospective DMR analyses in cord blood

At the age of 3, the main model showed evidence for DMRs in cord blood, annotated to genes *STK32C* and *MIR886*, being associated with internalising problems. Only the association with the *STK32C* annotated region remained after adjustment for maternal anxiety and depressive symptoms during pregnancy (Table 3). In the sex‐stratified models, no regions were identified in the male stratum, but three regions were identified in the models restricted to females. One of these regions (at *MIR886* (*VTRNA2‐1*)) was the same as the region identified in the unstratified analyses using the complete group. The other two regions are annotated to the genes *PON3* and *PPT2; PRRT1* (Table 3). At the age of 6, one region in cord blood, that is annotated to gene *PFKFB2*, was associated with internalising problems (Table 3). This region was also differentially methylated in the model additionally adjusted for maternal anxiety and depression symptoms during pregnancy. Each of the sex strata showed associations with two different DMRs in cord blood. In females, the regions are annotated to genes *MIR548F5, NBEA, MAB21L1* and *SLC1A2*. In males, only one of the regions was annotated to a gene (*ADSSL1*; Table 3). None of the internalising problems‐associated DMRs in cord blood were overlapping between ages 3 and 6 years (Table 3).

#### Cross‐sectional DMR analysis in peripheral blood

In the cross‐sectional analysis, the main model showed evidence for two DMRs in peripheral blood, mapped to genes *C10orf26* and *FAM125A*, being associated with internalising problems at age 6. The model adjusted for maternal anxiety and depression indicated an internalising problem‐associated DMR annotated to gene *FAM84A* (Table 3). The sex‐stratified analyses showed evidence for 5 DMRs in the female (annotated genes *MIR2110* (*C10orf118*), *HMGB1*, *FBXO21* and *KCNJ9*) and 1 DMR in the male models (annotated gene *HOXA4*) to be associated with internalising problems at age 6.

### Database look‐up

A summary of the mental health phenotypes that appeared in the database search of the differentially methylated CpG sites and regions, and their corresponding annotated genes, can be found in Table 4. For an overview of all associated phenotypes, please refer to Appendix S8 (GeneCards), Appendix S9 (GWAS catalogue) and Appendix S10 (EWAS catalogue).

**Table 4:** Result overview of the database look‐up

Gene	Mental health phenotype	Data source
Prospective analysis: Internalising problems age 3 ~ Cord‐blood DNAm (*n* = 3,011)		
*STK32C*	Adolescent depression (DNAm studies), risk‐taking behaviour, ADHD, trauma exposure	GeneCards, GWAS, EWAS
*MIR886* (*VTRNA2‐1*)	Response to antidepressants, bipolar disorder, schizophrenia, borderline personality disorder, ADHD	GWAS Catalogue
*PON3, PPT2, PRRT1*	No links with mental health phenotypes	GeneCards, GWAS catalogue, EWAS catalogue
Prospective analysis: Internalising problems age 6 ~ Cord‐blood DNAm (*n* = 1,601)		
cg26668632	No links with mental health phenotypes	EWAS Catalogue
*IFNGR1*	Child abuse	EWAS Catalogue
*PFKFB2*	Schizophrenia, externalising behaviours	GWAS Catalogue
*SLC1A2*	Schizophrenia	GWAS Catalogue
*NBEA*	Antipsychotic treatment response in schizophrenia, post‐traumatic stress disorder, externalising behaviour	GWAS Catalogue
*ADSSL1*	Schizophrenia, childhood abuse	EWAS Catalogue
*MIR548F5, MAB21L1, SLC1A2*	No links with mental health phenotypes	GeneCards, GWAS catalogue, EWAS catalogue
Cross‐sectional analysis: Internalising problems age 6 ~ Childhood DNAm age 6 (*n* = 1,121)		
*C10orf26* (*WBP1L*)	Schizophrenia, anxiety disorder, worry, nap duration in severe depression	GWAS Catalogue
*KCNJ9*	Depression, schizophrenia	GWAS Catalogue, EWAS Catalogue
*MIR2110*	Schizophrenia, childhood abuse	EWAS Catalogue
*FBXO21*	Insomnia	GWAS Catalogue
cg07283896, cg08884410	No links with mental health phenotypes	EWAS Catalogue
*FAM84A* (*LRATD*)	Sex‐dependent genetic interactions, schizophrenia, bipolar disorder, major depressive disorder	GWAS Catalogue
*FAM125A* (*MVB12*), *HOXA4, HMGB1*	No links with mental health phenotypes	GeneCards, GWAS cataligue, EWAS catalogue

#### Prospective analysis in cord blood

The differentially methylated CpG site (cg26668632, annotated to *IFNGR1*) had no reported mental health associations, though another *IFNGR1* site (cg13370280) was previously linked to childhood abuse (EWAS catalogue).

*STK32C* was previously associated with adolescent depression (GeneCards) and linked to risk‐taking, ADHD and trauma exposure (GWAS catalogue), with additional ADHD‐related methylation findings (EWAS catalogue). *MIR886* (*VTRNA2‐1*) showed GWAS catalogue associations with several psychiatric phenotypes, but no links in GeneCards or the EWAS catalogue. *PON3* and *PPT2/PRRT* showed no associations across databases. No mental health associations for *IFNGR1* were found in GeneCards or the GWAS catalogue. *PFKFB2* was associated with schizophrenia and externalising behaviours (GWAS catalogue), with no links in the other databases. *SLC1A2* and *NBEA* were linked to schizophrenia and other psychiatric phenotypes (GWAS catalogue). *ADSSL1* had no associations in GeneCards or the GWAS catalogue, but EWAS catalogue linked DNAm at CpGs annotated to *ADSSL1* with schizophrenia and child abuse. *MIR548F5* and *MAB21L1* showed no associations across databases. A comprehensive summary of these associations is provided in Table 4.

#### Cross‐sectional analysis in peripheral blood

No mental health‐related associations were reported in the EWAS Catalogue for the differentially methylated CpG sites cg07283896 and cg08884410, both of which lacked gene annotations.

*C10orf26* (*WBP1L*) showed links with schizophrenia, anxiety disorder, worry and nap duration in severe depression in the GWAS catalogue. *FAM84A* (*LRATD*) showed associations with sex‐dependent genetic interactions related to schizophrenia, bipolar disorder and major depressive disorder in the GWAS Catalogue. DNAm at *MIR2110* has been associated with schizophrenia and childhood abuse in the EWAS catalogue, though no associations appeared in the GWAS Catalogue or GeneCards. *KCNJ9* was associated with depression (GWAS Catalogue) and schizophrenia (EWAS Catalogue). *FBXO21* was linked to insomnia in the GWAS Catalogue. No mental health‐related associations were reported for *FAM125A* (*MVB12*), *HOXA4* or *HMGB1* across any of the examined databases. A detailed overview of these associations is presented in Table 4.

## Discussion

### Summary and interpretation of findings

The results of this study provide some evidence for a link between DNAm and internalising problems in childhood. Although the probe‐level analyses did not identify DNAm at single probes to be associated with childhood internalising problems, the higher‐powered regional‐based analyses revealed several internalising problems‐associated DMRs in cord and peripheral blood.

Results differed depending on the timing of assessments, both for DNAm (cord blood at birth vs. peripheral blood in childhood) and for internalising problems (assessed at ages 3 and 6 years). In cord blood, two DMRs were associated with internalising problems at age 3 (annotated to *STK32C*, *MIR886*) and one at age 6 (*PFKFB2*). In peripheral blood, two DMRs (*C10orf26* (*WBP1L*), *FAM125A*) were associated with internalising problems at age 6. This aligns with a study from the PACE consortium, which found associations specific to the timing of DNAm assessment (birth vs. childhood) for most outcomes, such as ADHD and general psychopathology (Neumann et al., 2025).

Two non‐exclusive explanations may account for this pattern: (1) measurement artefacts, such as differences in statistical power across analyses due to varying sample sizes, distinct confounding structures at birth versus childhood and tissue/composition differences between cord and peripheral blood (cell composition, gene regulation differences), which may affected detection (Martino et al., 2011; Mulder et al., 2020). Consistent with this, DNAm patterns were more similar within the same tissue across ages than across tissues at the same age: cord‐blood DNAm showed higher correlation between the age 3 and age 6 analyses than did cord versus peripheral blood compared at age 6 (Figure S14). (2) It may indicate true developmental specificity: DNAm marks and internalising problems likely relate in an age‐dependent manner as the immune system and emotion‐regulation circuitry mature, postnatal environments accumulate (e.g. parenting, health practices, socioeconomic context) and symptom profiles reorganise from ages 3 to 6. Internalising problems were moderately correlated from ages 3 to 6 (*r* = .39–.56; Appendix S5, Figure S1), suggesting both continuity and developmental reorganisation in symptom profiles across early childhood.

Except for the association between DNAm at *STK32C* and internalising problems at age 3, no congruence in results was observed between the maternal anxiety and depression adjusted and unadjusted models, indicating that maternal anxiety and depression may play a more complex role in shaping DNAm patterns (e.g. conditioning on variance indexed by maternal prenatal anxiety/depression, such as familial liability, correlated prenatal exposures and shared‐informant effects). The association between DNAm at *MIR886* (*VTRNA2‐1*) and child internalising problems at age 3 came up only after adjusting for maternal depression. As *MIR886* (*VTRNA2‐1*) is 75% maternally imprinted and has previously been linked to perinatal factors, such as maternal age and season of conception (Carpenter et al., 2018), this locus may reflect sensitivity to the broader prenatal environment. However, interpretations should remain cautious, as these associations likely reflect complex interactions between biological, environmental and social factors rather than direct causal pathways. Further research is needed to understand the functional effects of the DMRs in a developmental context and clarify timing effects (Neumann et al., 2025).

The database search indicated that several genes mapping to our DMRs have prior links to mental health phenotypes. For example, *STK32C*—implicated here via cord‐blood DNAm in relation to age 3 internalising problems—has been reported in adolescent depression (Dempster et al., 2014), and a related family member, *STK32B*, showed differential DNAm among adolescents at risk for generalised anxiety disorder (Ciuculete et al., 2018). In addition, annotation resources (GWAS Catalogue, GeneCards) list associations for other DMR‐mapped genes with depression (*MIR886* [*VTRNA2‐1*], *KCNJ9*, *FAM84A* [*LRATD1*]; Table 4) and anxiety disorders (*C10orf26* [*WBP1L*]). We do not interpret these cross‐references as locus‐level replication, given differences in age, tissue and phenotype; rather, they provide convergent, hypothesis‐generating context suggesting that pathways tagged by our DMRs overlap with those implicated in later internalising‐related phenotypes.

Sex‐stratified analyses revealed more DNAm associations with internalising problems in females, despite no observed sex differences in internalising problems in our study (Table 1). These findings should be interpreted with caution, as the direction of the probe‐level effects was the same for both sexes, and the female group was slightly larger with lower variability (Appendix S5, Table S2). Prior research highlights sex differences in autosomal DNAm and suggests sex‐specific epigenetic signatures could inform precision psychiatry (Grant, Wang, Kumari, Zabet, & Schalkwyk, 2022; Mulder et al., 2021; Müller et al., 2021; Tesfaye et al., 2024; Xia et al., 2021). Yet, it is unclear whether DNAm sex differences are due to different biological pathways, socialisation or both (Gutman & Codiroli McMaster, 2020).

### Strengths and limitations

This investigation of DNAm and internalising problems in very young children complements existing evidence, which mainly comprises adult populations (Barker, Walton, & Cecil, 2018). The inclusion of two different DNAm assessment time points (cord and peripheral blood) enabled capturing prenatal and postnatal risk factors. The investigation of internalising problems at age 3 and age 6 allowed evaluating the persistence of these effects across development. Another strength is in the application of an epigenome‐wide probe‐level and regional analysis within a multi‐cohort framework, in a hypothesis‐free way, which may reveal novel biological pathways to anxiety and depression. Last, the relatively large sample size and prospective design helped indicate the temporal sequence of events and reduced recall bias.

The results of this study should be interpreted considering the following limitations. Statistical power was compromised by the high zero inflation of children's internalising problems at both time points. The demographic profile suggests children were likely at low risk for adverse experiences, and thus, it is unclear whether results generalise to less advantaged populations. Selection bias is common in longitudinal cohort studies (Boyd et al., 2013; Taylor et al., 2018) and families with a less advantaged background and more mental health problems are more likely to drop‐out (Magnus et al., 2016; Wolke et al., 2009). This bias may be stronger for the DNAm subsamples, which are commonly selected based on completeness of other cohort variables (Relton et al., 2015). We relied on maternal‐report questionnaires because this informant was available consistently across cohorts and ages for harmonisation, but multi‐informant and repeated assessments (e.g. teacher and later self‐report) could reduce rater‐specific bias and better capture context‐specific symptoms; however, such data are often not uniformly available across cohorts and assessment time points. The database search may be influenced by the research focus on certain phenotypes, such as schizophrenia, which has been comprehensively studied in psychiatric genetics (Zakaria et al., 2023). Last, this study relied on DNAm assessed in blood, yet the tissue of most interest for mental health phenotypes is brain tissue. There is some evidence that part of blood DNAm can function as a proxy for brain DNAm (Davies et al., 2012; Kaminsky et al., 2009; Walton et al., 2016). Regardless of tissue concordance, these results may contribute to developing DNAm biomarkers for early detection of internalising problems (Walton, Relton, & Caramaschi, 2019).

### Future research

Future EWAS with larger sample sizes are needed to clarify the relationship between DNAm and internalising problems. The higher‐powered regional analysis found more evidence for associations between DNAm and internalising problems, warranting replication by independent studies. Future research using methylation profile scores could complement this study's approach by leveraging subtle changes in DNAm across the genome (Thompson et al., 2022). Methylation profile scores summarise DNAm across many CpG sites into a single, individual‐level score (typically a weighted sum based on prior EWAS results), rather than testing one CpG (or one region) at a time. They can be useful when DNAm differences are expected to be small and distributed across the methylome—that is when there may be many small effects that do not reach epigenome‐wide significance individually but can add up to a detectable signal when aggregated (Thompson et al., 2022). Studies should invest in more detailed phenotyping and richer assessment of anxiety and depression in children, for instance include multiple assessment time point and informants, improved contextual coverage across settings (home vs. school), ideally repeated over time to differentiate transient from persistent difficulties (Dunn et al., 2019; Sallis et al., 2019). Where feasible, integrating multi‐informant reports (e.g. parents, teachers and self‐report when age‐appropriate) would reduce rater‐specific bias and improve contextual coverage, although informant availability and instruments often differ across cohorts and waves, making cross‐cohort harmonisation challenging. This aligns with broader calls for precision behavioural phenotyping to reduce heterogeneity and strengthen biology–psychopathology associations (Tiego et al., 2023).

Also neuroimaging phenotypes may be included, which may serve as intermediate phenotypes for mental health problems (Walton et al., 2023). Methods like longitudinal mixed models or Structured Life Course Modelling Approach could help to capture developmental trajectories. Replication in more diverse populations with higher risks for adverse early‐life experiences is necessary, and more extensive research is needed to understand the observed sex differences and their clinical significance. Investigation of gender factors, such as caregiver expectations and gendered stress, could help understand how sociocultural factors shape DNA methylation and mental health (Cattaneo et al., 2024). Future research might explore genotype–DNAm interactions, as certain genotypes may be more susceptible to environmental effects (Brummelte, Mc Glanaghy, Bonnin, & Oberlander, 2017; Oberlander et al., 2010; Olsson et al., 2010; Teh et al., 2014). Additionally, future studies should shift focus from early adverse experiences to exploring resilience factors in children, examining how positive early‐life experiences affect DNAm and mental health (Kentner, Cryan, & Brummelte, 2019).

## Conclusion

The findings of this study provide some evidence for associations between DNAm and childhood internalising problems, with distinct signals observed at different assessment time points for both DNAm (birth vs. childhood) and internalising problems (at ages 3 and 6). Despite the high zero inflation of internalising problems, the DMR analyses suggested differences in DNAm at regions annotated to genes, which have previously been associated with mental health phenotypes related to internalising problems. It is likely that these effects would be further exacerbated in populations with more occurrences of internalising problems. As anxiety and depression are highly polygenic disorders, which are likely influenced by the interplay of small effects of many different genes, more EWAS are needed to replicate or invalidate findings of this study, as well as to detect novel differentially methylated regions, which might have been missed.

## Ethical considerations

For ALSPAC, ethical approval was obtained from the ALSPAC Ethics and Law Committee and the Local Research Ethics Committees under approval references E1808 (28 November 1989), 49/89 (5 April 1990) and 90/8 (28 June 1990). Informed consent for data collection was obtained from participants in accordance with the ethical standards in place at the time of recruitment and follow‐up (http://www.bristol.ac.uk/alspac/researchers/research‐ethics/). For the Generation R Study, all phases of the study were approved by the Medical Ethics Committee of Erasmus MC, University Medical Center Rotterdam, the Netherlands, including approvals granted in 2001 (MEC‐198.782.2001.31), 2002 (217.595.2002.202), 2007/2008 (MEC‐2007‐413_NL21545.078.08), 2012 (MEC‐2012–165_NL40020.078.12, version 03) and 2015 (MEC‐2015‐749_NL55105.078.15), as well as 2024 (NL‐OMON55374). Written informed consent was obtained from all participating mothers, and from age 12 onwards, children also provided written consent in accordance with Dutch law. For child participants, parental consent was obtained. Participants received written and oral information prior to each study phase and could refuse participation in specific measurements. MoBa is regulated by the Norwegian Health Registry Act. The current study was approved by the Regional Committees for Medical and Health Research Ethics nr 2020/ 185800 in January 2020. Informed consent has been attained for all participants and was approved by the Regional Committees for Medical and Health Research Ethics, before the study was initiated. At enrolment, mothers provided informed consent for themselves and their children. At age 18 years, the children become independent participants and receive an information letter about their rights, including how to withdraw.

**Key points**

**What's known?**

- Childhood internalising problems are influenced by genetic, prenatal and postnatal environments and are risk factors for later depression.
- DNA methylation (DNAm) has been proposed as a potential biological mechanism linking early exposures to mental health outcomes, though evidence remains inconsistent.

**What's new?**

- This meta‐analysis examined associations between child blood DNAm and internalising problems at multiple time points using both probe‐ and region‐level approaches.
- Region‐level analyses, rather than individual CpG sites, revealed associations with internalising problems, identifying regions annotated to genes previously linked to psychiatric phenotypes.

**What's relevant?**

- While findings are preliminary, they suggest that DNAm signatures in early life may help identify biological pathways relevant to childhood internalising problems.
- Larger, longitudinal studies with detailed phenotyping are warranted to confirm these associations before translating DNAm findings into clinical risk markers or interventions.

## Supporting information


**Appendix S1:** Avon Longitudinal Study of Parents and Children (ALSPAC).
**Appendix S2:** MoBa.
**Appendix S3:** Generation R.
**Appendix S4:** Methods.
**Appendix S5:** Results.
**Table S1:** Correlations between covariates and child internalising problems and mean internalising problems by maternal smoking status across cohorts.
**Figure S1:** Correlation matrix of the cord‐blood probe‐level meta‐analysis results: Internalising problems age 3.
**Figure S2:** Correlation matrix of the cord blood probe‐level meta‐analysis results: Internalising problems age 6.
**Figure S3:** Correlation table of internalising problems and covariates.
**Figure S4:** Correlation matrix of the cross‐sectional meta‐analysis results: Internalising problems age 6.
**Figure S5:** QQ plot of the cord‐blood probe‐level meta‐analysis results: Internalising problems age 3.
**Figure S6:** QQ‐plot of the cord blood probe‐level meta‐analysis results: Internalising problems age 6.
**Figure S7:** QQ‐plot of the cross‐sectional meta‐analysis results: Internalising problems age 6.
**Figure S8:** Manhattan and QQ plots of the covariate adjusted models across time points.
**Figure S9:** Manhattan and QQ plots of the covariate adjusted female‐sex stratified models across time points.
**Figure S10:** Manhattan and QQ plots of the covariate adjusted male‐sex stratified models across time points.
**Figure S11:** Manhattan and QQ plots of the maternal anxiety/depression adjusted models across time points.
**Table S2:** A summary of results of each EWAS model from the probe‐level analysis.
**Figure S12:** Leave‐one‐out plot of the cord‐blood meta‐analysis age 7 (Cg26668632).
**Figure S13:** Leave‐one‐out plot of the childhood peripheral‐blood meta‐analysis age 7 (Cg08884410).
**Figure S14:** Leave‐one‐out plot of the childhood peripheral‐blood meta‐analysis age 7 (Cg07283896).
**Table S3:** Correlations between covariates and child internalising problems and mean internalising problems by maternal smoking status across cohorts.
**Appendix S6:** Cohort‐specific acknowledgments and funding.
**Appendix S7:** Author‐specific funding statements.

**Appendix S8:** GeneCards.

**Appendix S9:** GWAS catalogue.

**Appendix S10:** EWAS catalogue.

## Data Availability

The datasets analysed in this study are available from the corresponding cohort data access committees upon reasonable request.
